# The *Arabidopsis thaliana* trehalose-6-phosphate phosphatase gene *AtTPPI* regulates primary root growth and lateral root elongation

**DOI:** 10.3389/fpls.2022.1088278

**Published:** 2023-01-13

**Authors:** Qingfang Lin, Jiaxin Gong, Zhiliang Zhang, Zizi Meng, Jianyong Wang, Song Wang, Jing Sun, Xu Gu, Yuting Jin, Tong Wu, Nuo yan, Yuxin Wang, Lei Kai, Jihong Jiang, Shilian Qi

**Affiliations:** ^1^ School of Life Sciences, Jiangsu Normal University, Xuzhou, Jiangsu, China; ^2^ Institute of Genetic and Developmental Biology, Chinese Academy of Sciences, Beijing, China; ^3^ Lushan Botanical Garden, Chinese Academy of Sciences, Jiujiang, Jiangxi, China; ^4^ Technical Services and Sales Department, Zhengzhou Xuanyuan Biotechnology Co. LTD, Zhengzhou, Henan, China

**Keywords:** Arabidopsis, AtTPPI, primary root growth, auxin, lateral root elongation

## Abstract

Roots are the main organs through which plants absorb water and nutrients. As the key phytohormone involved in root growth, auxin functions in plant environmental responses by modulating auxin synthesis, distribution and polar transport. The *Arabidopsis thaliana trehalose-6-phosphate phosphatase* gene *AtTPPI* can improve root architecture, and *tppi1* mutants have significantly shortened primary roots. However, the mechanism underlying the short roots of the *tppi1* mutant and the upstream signaling pathway and downstream genes regulated by *AtTPPI* are unclear. Here, we demonstrated that the *AtTPPI* gene could promote auxin accumulation in *AtTPPI*-overexpressing plants. By comparing the transcriptomic data of *tppi1* and wild-type roots, we found several upregulations of auxin-related genes, including *GH3.3*, *GH3.9* and *GH3.12*, may play an important role in the *AtTPPI* gene-mediated auxin transport signaling pathway, ultimately leading to changes in auxin content and primary root length. Moreover, increased *AtTPPI* expression can regulate primary root growth and lateral root elongation under different concentration of nitrate conditions. Overall, constitutive expression of *AtTPPI* increased auxin contents and improved lateral root elongation, constituting a new method for improving the nitrogen utilization efficiency of plants.

## 1 Introduction

In nature, unlike animals, plants are sessile organisms. Plants must be able to alter their growth and development to adapt to various environmental conditions through multiple means at any time. Roots, which are major plant organs, play an important role in absorbing water and acquiring nutrients from the soil (Petricka et al., 2012). Plant growth and development are tightly regulated, and root system architecture is modulated by many factors (Malamy, 2010). Numerous studies have shown that various hormones function in root development. Hormones are small molecules essential for plant growth. Many hormones function when plants encounter various environmental conditions and act as internal cues to alter plant development.

Hormones involved in root development include auxins, cytokinins (CKs), brassinosteroids (BRs), gibberellins (GAs), abscisic acid (ABA), ethylene, salicylic acid (SA), jasmonates (JAs) and strigolactones (SLs) (2018; Mariana and Javier, 2011; Garay-Arroyo et al., 2012; Ding and De Smet, 2013; Paponov et al., 2013; Shani et al., 2013; Wei and Jia, 2016; Huang et al., 2017; Olatunji et al., 2017; Xu et al., 2020). Among the majority of hormones, auxin is the fundamental and most researched hormone, and it has been recognized as the key regulator that modulates primary root growth (Petricka et al., 2012). Auxin participates in the regulation of plant growth and development by modulating its own synthesis, distribution and polar transport (Petricka et al., 2012; Wang and Jiao, 2018). Auxin distributed as a gradient in plants, decreasing sequentially from the root tip, meristematic zone and zone of elongation (2014; Sabatini et al., 2006; Petersson et al., 2009; Band et al., 2014). The growth of roots mainly depends on cell elongation and meristematic activity (Petricka et al., 2012). Therefore, auxin distribution and transport are both fundamental and critical factors for root growth. The known members responsible for the polar transport of auxin in plants mainly include auxin influx carriers AUXIN1 (AUX1)/LIKE-AUXs (LAXs) and PIN-FORMED (PIN) auxin efflux transporter family members (Zazimalova et al., 2010; Murphy, 2011). PIN genes encode transmembrane protein that has been reported to affect primary root length by affecting polar auxin transport. Interestingly, the effect of PINs on plant primary roots is bidirectional; that is, a high concentration inhibits root growth, and a low concentration promotes root growth (Keek et al., 2009; Adamowski and Friml, 2015; Sascha et al., 2020). Therefore, in plants, there must be a suitable range of auxin concentrations that promote elongation of the primary root. How do plants sense the external environment or internal signals to regulate their root growth?

Apart from plant hormones, the nutrition especially mineral element in soil is also a main effector influencing root architecture. Nitrogen (N), as the mineral element most demanded by plants, its limitation would decrease crop yield worldwide. Under the condition of limited soil nutrients, how to improve the utilization rate of nitrogen has long become the focus of attention. Fortunately, plants also evolved various mechanisms to respond N limitation, among them, changing the root system architecture is the main strategy (Nibau et al., 2008). Reports have proved that root nitrate (NO^3–^) uptake characteristics involved in N avaibility (Lejay et al., 1999). Nitrate mainly stimulates lateral root (LR) elongation; lateral roots are particularly important in root systems, it play a crucial role in adapting to various environmental conditions as important organs for plants to absorb water, nutrients and cope with multiple stress (Casimiro et al., 2003; Nibau et al., 2008; Petricka et al., 2012). While, the lateral root development is also closely related to the distribution of auxin, auxin-dependent LR initiation have been identified (Casimiro et al., 2003). Since the auxin and nitrogen both effluence the LR development, is there a cross-talk between their transduction pathway?

Our previous research on the Arabidopsis trehalose-6-phosphate phosphatase (*TPP*) gene *AtTPPI* showed that auxin transport in the *tppi1* mutant was compromised, and the result was caused by decreased PIN1 and PIN3 protein levels (Lin et al., 2020). In addition, it is likely that the decreased auxin transport caused the shortened primary roots observed. However, there have been no in-depth studies on the relationships between auxin and *AtTPPI* genes, and the upstream genes modulating the auxin signaling pathway and the downstream genes regulated by the *AtTPPI* gene are not clear. Here, we report that the Arabidopsis *TPP* gene *AtTPPI* was capable of rescuing the short primary root length of the *tppi1* mutant. Overexpression of *AtTPPI* increased the auxin level. Our results also found that overexpression of the *AtTPPI* gene can promote the growth of primary roots and increase the lateral root number under high nitrogen conditions. These findings suggest that *AtTPPI* mediates primary root growth regulation by positively regulating auxin levels and that *AtTPP1* may play a crucial role in nitrogen acquisition.

## 2 Material and methods

### 2.1 Plant materials and growth conditions

The *Arabidopsis thaliana* wild type (WT) used in this study was the Columbia-0 (Col-0) ecotype. It was purchased from ABRC. The two overexpression lines OE4 and OE5 were the same as the *35S:AtTPPI* transgenic lines mentioned previously (Lin et al., 2020). The seeds were all in our library from Jiangsu Normal University. We ensure that our experimental research on plants; including the collection of plant material, comply with relevant institutional, national and international guidelines and legislation.

The seeds of these lines were surface sterilized and sown vertically on Murashige and Skoog (MS) media (supplemented with 1% sucrose and 6 g L^–1^ agar) and stored at 4°C for three days. Then, all the plates were transferred to a plant tissue culture chamber under a 16 h light/8 h dark photoperiod, a 22°C temperature, a 120 μmol quanta m^−2^ s^−1^ light intensity and 50% relative humidity.

### 2.2 Root length assay

For the vertical MS media culture, the plates were all placed in a plant tissue culture chamber, and after 14 days of growth, images were taken. Afterward, the roots were removed and measured one by one.

For soil culture, sterile seeds were first sown on MS media (supplemented with 1% sucrose and 6 g L^–1^ agar), after which they were placed in a 4°C refrigerator for three days to ensure uniform germination and allowed to grow at 22°C under long days (16 h light/8 h dark photoperiod) for 7 days. Then, the seedlings were transplanted into soil, with one seedling per pot. After 14 days of growth, images were taken, and rosette leaves and root lengths were measured separately.

### 2.3 Determination of auxin contents

Auxin contents were measured by the indoleacetic acid (IAA) and auxin enzyme-linked immunosorbent assay (ELISA kit, RXJ1401005PL, Quanzhou Ruixin Biological Technology Co., LTD) kits following the manufacturer’s protocol. For sample collection, the samples were ground in 1 mL of 80% (v/v) methanol. The extract was then centrifuged at 8000 rpm for 1 h; Afterward, the supernatant was passed through a C-18 column, and the details of the steps were as follows: washing with 80% (v/v) methanol, 100% (w/v) methanol, 100% (w/v) ether and then 100% (w/v) methanol. Afterward, the hormone fractions were dried and dissolved in 1 mL of phosphate-buffered saline (PBS; pH 7.4) for further analysis. After they were mixed, the samples were incubated at room temperature for 30 min and then centrifuged at 8000 rpm for 15 min at 4°C. The supernatant was then removed and stored temporarily at 4°C for later use.

For determination, the proteins were quantified using a protein quantification kit (RXKM0488-100, Quanzhou Ruixin Biological Technology Co., LTD) to facilitate the subsequent auxin content calculation. The determination of auxin was as follows. First, all the reagents were prepared, after which they were incubated at room temperature for 30 min. Washing solution was prepared according to the sample amount to be measured. A precoated plate was then taken, and a blank control without any liquid was also included. Two wells were designated for each 50 μL of calibration material, and 50 μL of sample material to be measured was to each other test well. Fifty microliters of biotinylated antigen were subsequently added to all the wells except the blank control well, and the contents of the wells were thoroughly mixed. The plates were then sealed with a membrane and incubated at 37°C for 60 min. For manual washing of the plates, the liquid in the wells was first discarded, after which each well was filled with washing liquid. Then plants were subsequently incubated at room temperature for 10 s, dried by swinging (which was repeated 3 times) and then patted dry. Chromogenic agent A (50 μL) and chromogenic agent B (50 μL) were added to each well. Afterward, the plates were shaken and the contents of the wells were thoroughly mixed; the plants were subsequently incubated at 37°C in the dark for 15 min. Then, 50 μL of stop solution was added to each well. A microplate reader was used to determine the absorbance at 450 nm in each well. Then, a standard curve equation was generated, with the standard concentration representing the horizontal coordinate and the corresponding absorbance (OD value) representing the vertical coordinate, using computer software. Four-parameter logistic curve fitting (4-pl), the sample absorbance (OD value), and the standard equation were then to calculate the sample concentration value. Three biological replicates of each hormone were included for this experiment.

### 2.4 RNA sequencing and analysis

Total RNA was extracted from the roots of vertically cultured 14-day-old seedlings using TaKaRa RNAiso Plus (9109, Takara). Two micrograms of RNA were used for library construction; each sample was replicated three times. The transcriptomic data set used in this study was obtained using the Illumina HiSeq platform, and 150-bp high-quality trimmed paired-end reads were generated. The trimmed reads were subsequently mapped to the reference genome sequence of Arabidopsis using HISAT2 (http://ccb.jhu.edu/software/hisat2/faq.shtml) with the default settings (Kim et al., 2015). Differentially expressed genes (DEGs) were analyzed using edgeR (http://bioinf.wehi.edu.au/edgeR/) (Smyth, 2010). Reads of the RNA-seq data were mapped to the Arabidopsis reference genome (The Arabidopsis Information Resource 10 (TAIR10)), and genes whose expression was more than twofold that of the WT (P < 0.05) were considered differentially expressed.

### 2.5 Primary root length and lateral root number and length statistic

MS519 used in the experiment was a total nitrogen medium containing ammonium and nitrate nitrogen, while MS531 was a nitrogen free medium without any form of nitrogen. Sterile seeds of WT plants, *tppi1* mutants and OE5 overexpression lines were sown vertically on MS media (MS519, Phytotech, USA; supplemented with 0.5% sucrose and 10 g L^–1^ agar) and MS nitrate media with different concentration of KNO_3_ (MS531, Phytotech USA; supplemented with 0, 10 μM, 100 μM, 1000 μM and 10 mM KNO_3_, 0.5% sucrose and 10 g L^–1^ agar). Then, the plates were all placed in a plant tissue culture chamber, and 12 or 14 days later, images were taken. The primary roots and lateral roots were then removed and measured one by one.

## 3 Results

### 3.1 *AtTPPI* can rescue the short-root phenotype of the *tppi1* mutant

**Figure 1:**
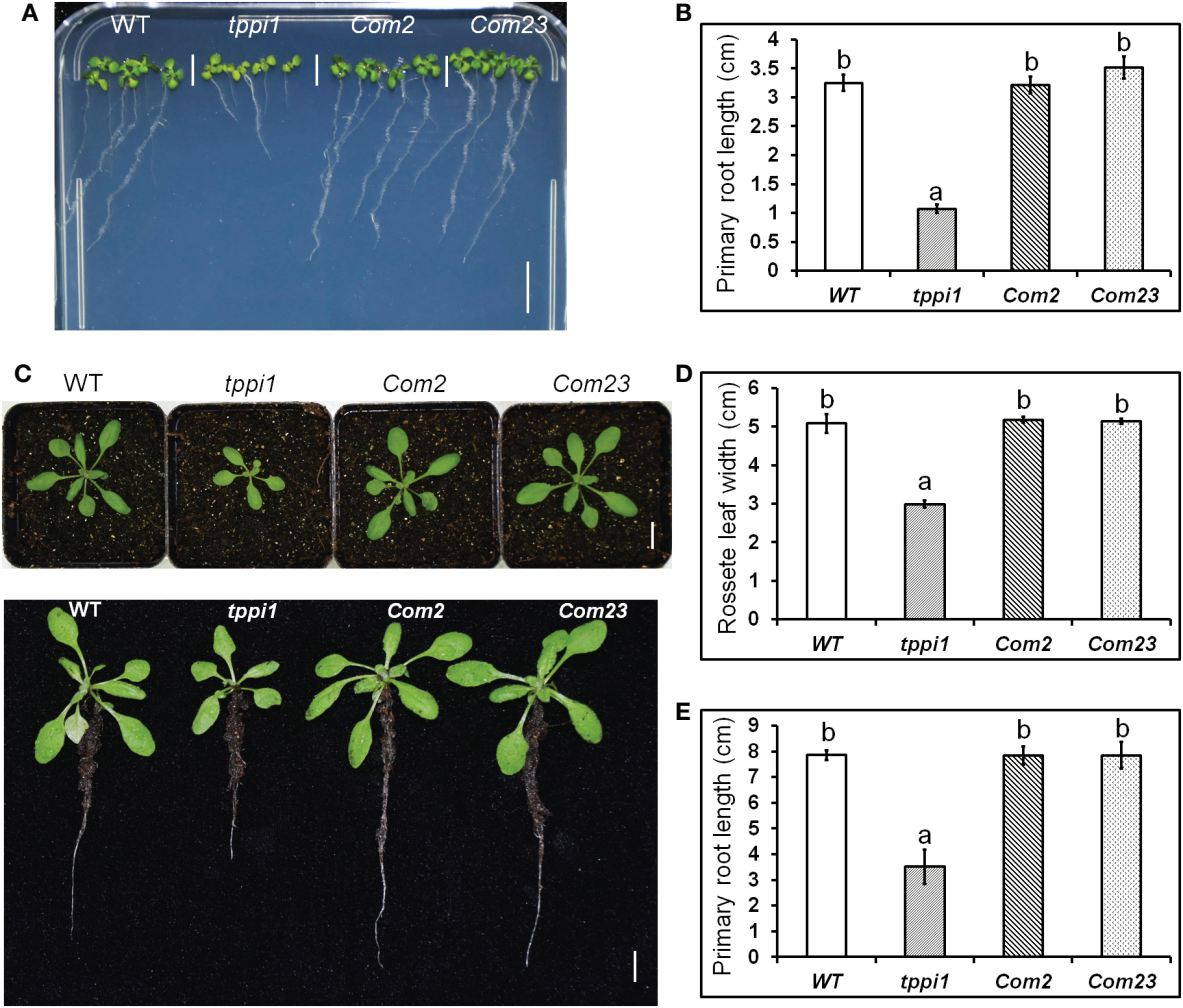
*AtTPPI* can rescue the short primary root of *tppi1* mutant. **(A)** The phenotype of primary root of WT, *tppi1* mutant, *com2* and *com23* on MS vertical medium for two-week days, Bar = 1 cm; **(B)** The statistical analysis of WT, *tppi1* mutant, *com2* and *com23* on MS vertical medium for two-week days. Three independent experiments were performed. Error bars indicate SEs (n = 3). Different lowercase letters **(A, B)** indicate significant differences among lines (Duncan’s test; p < 0.05); **(C)**. The phenotype of primary root of WT, *tppi1* mutant, *com2* and *com23* in 6×6 cm pot in soil, Bar = 1 cm; **(D, E)**. The statistical analysis of WT, *tppi1* mutant, *com2* and *com23* on rosette leaves size **(D)** and primary root length **(E)**. Error bars indicate SEs (n = 3). Different lowercase letters **(a, b)** indicate significant differences among lines (Duncan’s test; p < 0.05);.

Our previous study found that, compared with the WT, the *tppi1* mutant has a significantly shorter primary root (Lin et al., 2020). To verify whether this phenotype is caused by the *AtTPPI* gene, we introduced the same *AtTPPI* overexpression construct in which the gene was driven by the CaMV 35S promoter mentioned previously into the *tppi1* mutant. T3 homozygous lines were used for further root length assays. The results showed that the root length of the complementary lines (*Com2, Com23*) could be restored to that of the WT on the vertical MS media (**Figure 1**). Statistical analysis further suggested that the short-root phenotype of the *tppi1* mutant was indeed caused by the *AtTPPI* gene (**Figure 1B**).

We performed the same experiment in soil. One-week-old seedlings were transferred to 6 cm×6 cm pots filled with soil, and the phenotypes of the plants after 2 weeks of growth were imaged. Similar results were obtained as those above in MS culture (**Figure 1C**), and these were further confirmed *via* statistical analysis of the primary root length (**Figure 1D**). In addition, we measured the rosette leaf dimeter and statistically analyzed the data. The results showed that the rosette leaf size of *Com2* and *Com23* could also be restored to that of the WT (**Figure 1E**). This indicates that both the short-root phenotype and the small rosette size of the *tppi1* mutant were caused by the *AtTPPI* gene.

### 3.2 Overexpression of *AtTPPI* increases the auxin level in Arabidopsis

**Figure 2:**
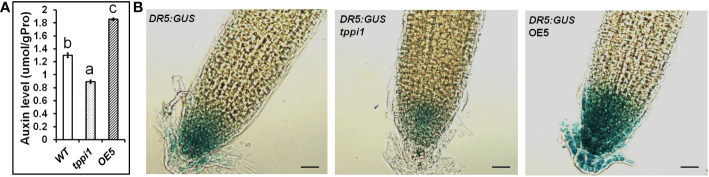
Elevated *AtTPPI* expression could enhance the auxin level. **(A)** The determination of auxin level in WT, *tppi1* mutant and OE5 overexpression plants. Error bars indicate SEs (n = 3). Different lowercase letters **(A–C)** indicate significant differences among lines (Duncan’s test; p < 0.05); **(B)** The GUS signal of WT, *tppi1* mutant and OE5 overexpression plants crossed with the *DR5:GUS* marker. Bar indicates 25 μm.

The GFP fluorescent signal of the *DR5rev:GFP* marker in the root tips of *tppi1* mutant and WT plants verified that the *tppi1* mutant has lower auxin contents than the WT plants do (Lin et al., 2020). To investigate the function of *AtTPPI* in terms of auxin, we further measured the auxin levels of WT plants, the *tppi1* mutant and overexpression line OE5 with an ELISA kit for auxin. The results showed that the overexpression line OE5 had a significantly increased auxin level compared with that of the WT (**Figure 2A**). In addition, the auxin level of the *tppi1* mutant was significantly lower than that of the WT, the results of which were consistent with the weaker *DR5rev:GFP* fluorescent signal in the mutant (Lin et al., 2020).

Meanwhile, we also crossed the *tppi1* mutant and overexpression line OE5 with *DR5:GUS* marker plants that showing auxin levels, respectively. The expression level of *DR5:GUS* in OE5 root was significantly higher than WT plants and *tppi1* mutant (**Figure 2B**). The result is similar with the above auxin content determination, which further indicate that the elevated *AtTPPI* expression indeed increase the auxin level.

### 3.3 Identification of *AtTPPI*-regulated genes in roots

**Figure 3:**
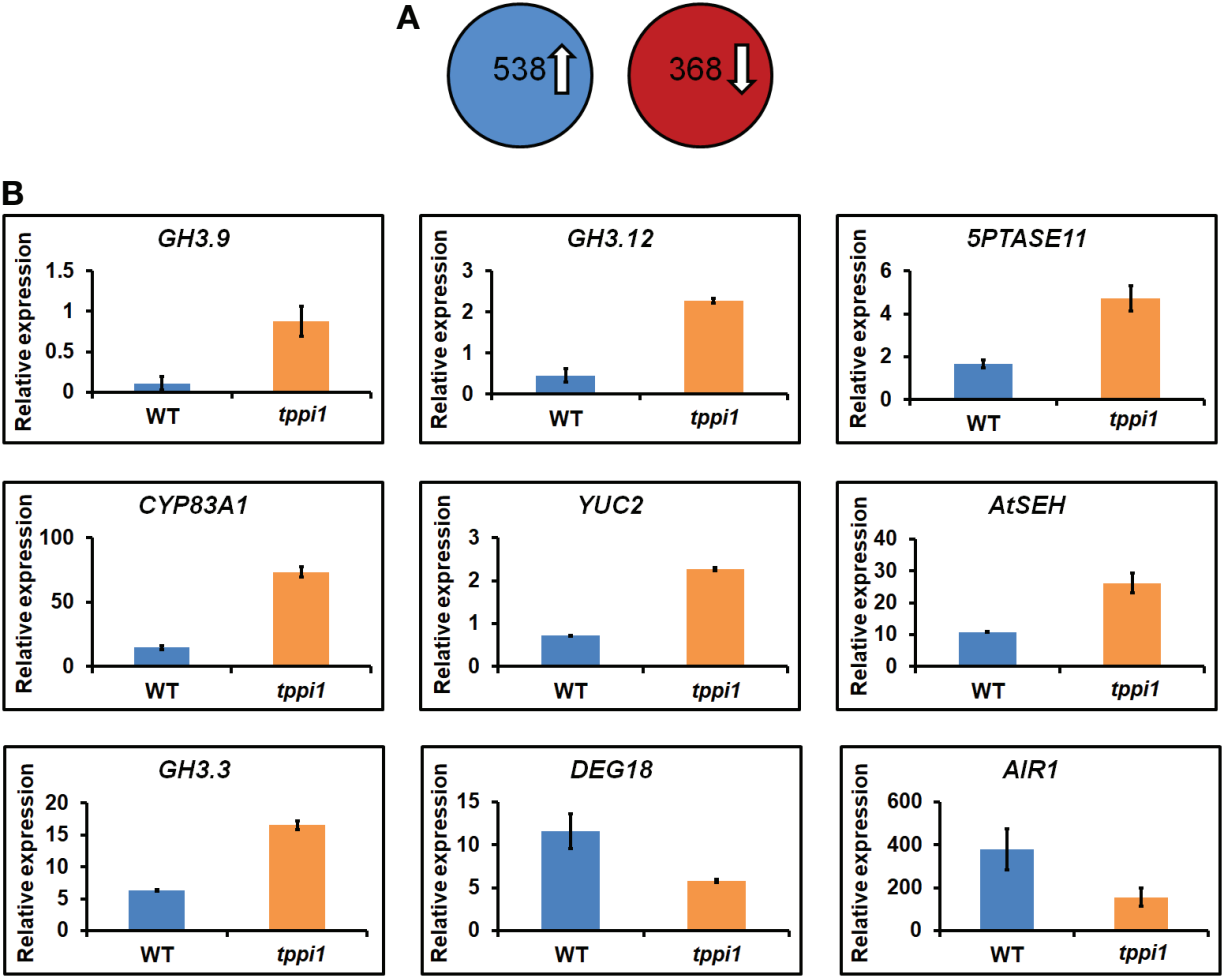
Transcriptome analysis of *tppi1* mutant and WT plants root. **(A)** The number of up-regulated and down-regulated DEGs; **(B)** Relative expression of nine auxin-related genes including seven up-regulated and two up-regulated genes in DEGs.

To further evaluate the role of *AtTPPI* in modulating the plant auxin response on a broader scale, we analyzed the transcriptomes of WT plants and the *tppi1* mutant. Total RNA of roots from 2-week-old seedlings growing vertically on MS media was isolated and subjected to RNA-seq (for which three biological replicates were included). A total of 3 Gb of clean data were obtained, which was mapped to the sequences of the gene models in the TAIR10 assembly (Smyth, 2010). The fragments per kilobase of transcript per million (FPKM) value of each gene were calculated for the WT and *tppi1* mutants. By using pairwise comparisons of the *tppi1* mutants and WT plants, we defined DEGs as those whose FPKM values changed by at least twofold (p value < 0.05) (Smyth, 2010). We then performed a comparative analysis of the WT and *tppi1* mutants. According to the screening conditions, 906 DEGs were identified; among these DEGs (*tppi1* vs. WT), 538 were upregulated, and 368 were downregulated (**Figure 3**). We expected that some of these genes would contribute to the significantly decreased primary root length of the *tppi1* mutants.

We therefore performed a gene functional annotation analysis of the 538 upregulated and 368 downregulated genes (**Supplementary Table S1**). Gene Ontology (GO) analysis revealed that the 538 and 368 genes were involved in various biological processes and molecular functions. We mainly focused on the DEGs related to the auxin response and found some important genes, including 7 upregulated genes and 2 downregulated ones (**Figure 3**). These important genes were reported to be involved in various processes in response to auxin. Interestingly, among the upregulated genes, there were three GH3 family genes identified, namely, *GH3.9*, *GH3.12* and *GH3.3*. Research on the function of *GH3* genes, which are primary auxin response gene family members, has mainly focused on auxin, including interactions with auxin and auxin response factors and interactions with plant defense responses mediated by plant signaling molecules such as SA (Staswick, 2005; Park et al., 2007; Wang et al., 2011; Weiste and Dröge-Laser, 2014). These genes are mainly involved in auxin inactivation (Staswick, 2005). Loss of function of the *GH3.9* gene has been reported to result in longer primary roots of Arabidopsis (Khan and Stone, 2007). Interestingly, the increased fold-change of *GH3.9* expression was the highest. The increased expression levels of the *GH3.9* gene in the *tppi1* mutant may contribute to the significantly shorter primary roots (**Figure 1**). In addition, apart from *GH3.9*, *GH3.12* has been reported to function in the defensive response, and loss of function of *GH3.12* resulted in decreased defense against pathogens, which suggested that the *AtTPPI* gene might function in plant defense responses.

**Figure 4:**
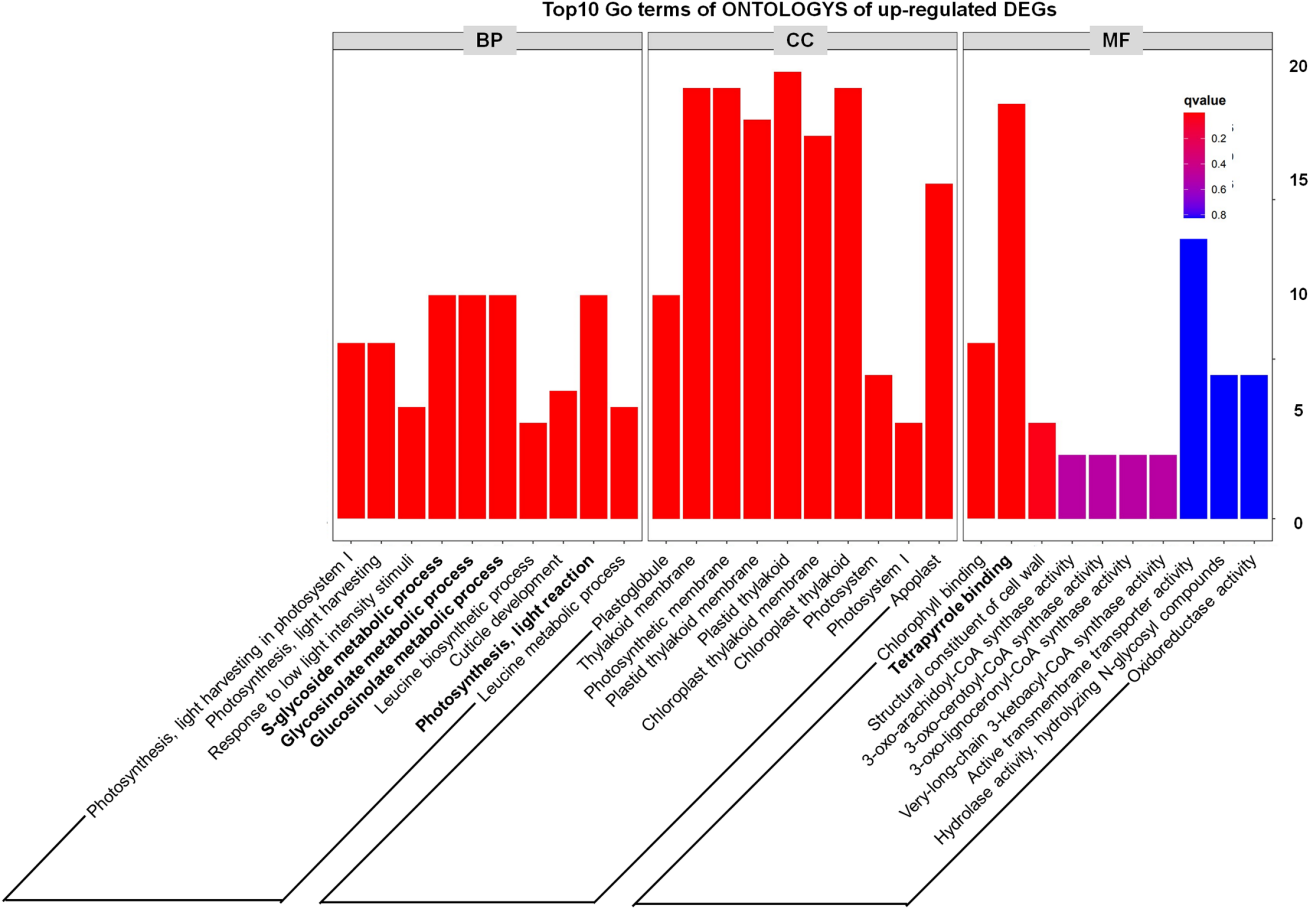
GO classification results of up-regulated DEGs.

In addition, through analyzing the Top10 GO terms of ONTOLOGYs of DEGs, we found that among the upregulated DEGs, “S-glycoside metabolic process, Glycosinolate metabolic process, Glucosinolate metabolic process and Photosynthesis, light reaction” were enriched (**Figure 4**), which indicate that the phenotype of *tppi1* mutant is likely connected with these processes. Our recent study also partly proved that sugar metabolism of *tppi1* mutant indeed changed, including increased sucrose, starch level and decreased glucose, which is likely responsible for the various development phenotype of *tppi1* mutant. Meanwhile, the molecular function responsible for the tetrapyrrole binding accounted for the most (**Figure 4**). Tetrapyrroles are linear or cyclic molecules containing four pyrrole rings, they usually function as cofactors in plants (Warren and Smith, 2009). In plants, apart from play role in photosynthesis and respiration, tetrapyrroles also participate in the assimilation of nitrogen/sulfur (Luo et al., 2020). Also, tetrapyrroles and tetrapyrrole-containing proteins have been reported to mediate the cellular detoxification of reactive oxygen species (Busch and Montgomery, 2015). The abundance of these genes suggests that *AtTPPI* may play a role in detoxification and nitrogen assimilation.

**Figure 5:**
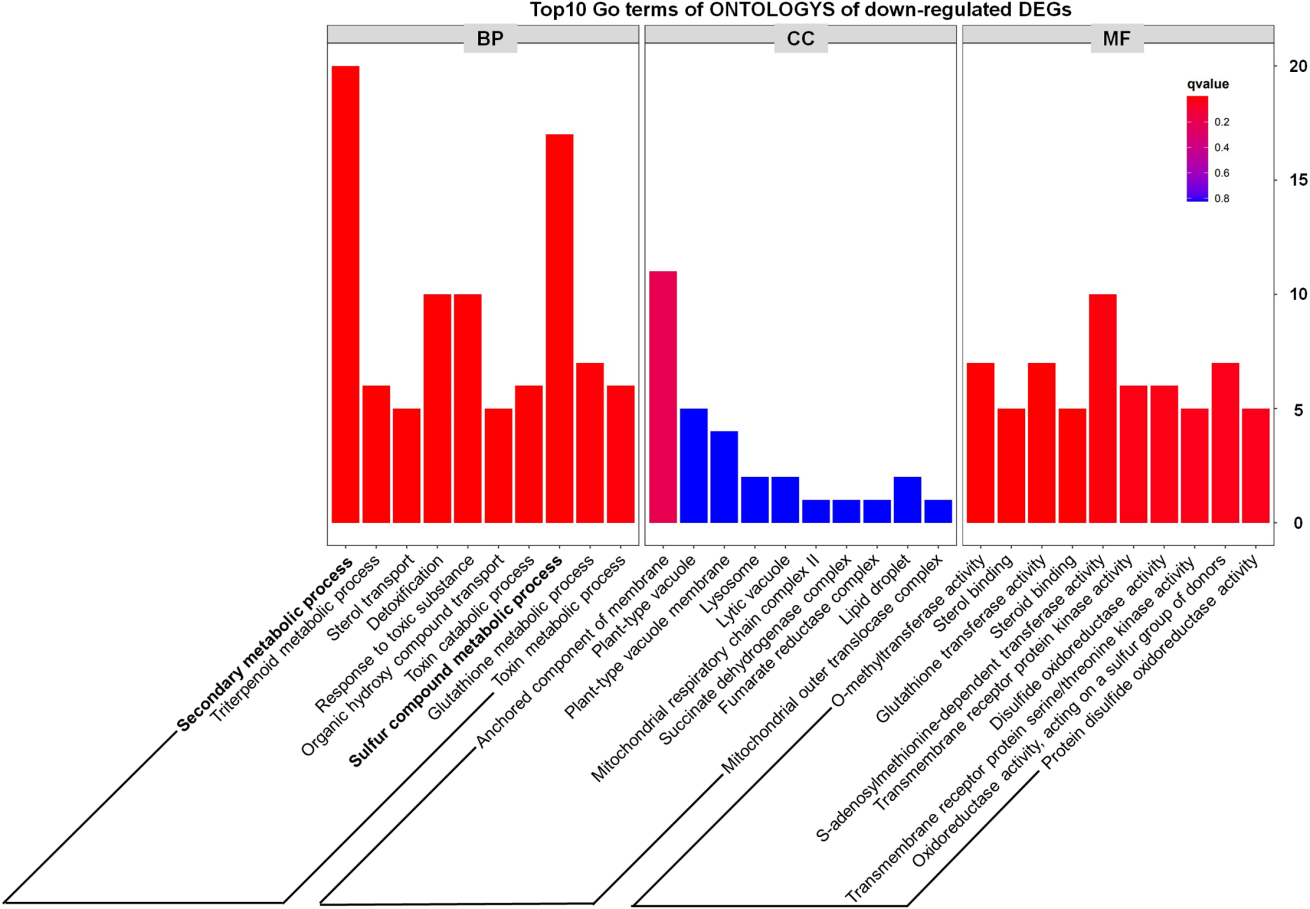
GO classification results of down-regulated DEGs.

In addition, we noted that metabolism-related genes including secondary metabolic process and sulfur compound metabolic process accounted for a large proportion of down-regulated DEGs (**Figure 5**), which preliminarily suggested that some metabolic pathways might be inhibited in the *tppi1* mutants. This is exactly consistent with the growth and development phenotype of *tppi1* mutant. For instance, *tppi1* mutant had significantly reduced rosette leaves, late-flowering, stunted root length, and numerous root hairs, shorten hypocotyl and delayed seed germination. In future, we will do more research about the relationship between the DEGs and the respective phenotype.

### 3.4 *AtTPPI* promote primary root growth, increase lateral root number under high nitrate conditions

Previous studies have suggested that LONG HYPOCOTYL 5 (HY5) plays a role in suppressing auxin signaling (Sibout et al., 2006; Cluis et al., 2004). HY5 is a basic domain leucine/zipper (bZIP) TF that is an important regulator that promotes photomorphogenesis and participates in the growth of plant roots, and this protein is regulated by light and hormones (Oyama et al., 1997; Toledo-Ortiz et al., 2014; Nawkar et al., 2017). Previous chromatin immunoprecipitation (ChIP) coupled to DNA chip hybridization (ChIP-chip) results also found that *AtTPPI* is an *in vivo* target of HY5 (Lee et al., 2007). Apart from its function in modulating auxin levels, HY5 is also involved in nitrogen metabolism and works to integrate the light-mediated nitrogen regulatory network (Chen et al., 2016; Gelderen et al., 2021). Nitrogen is an essential element in plant nutrition. Moreover, plants will preferentially absorb nitrate nitrogen (NO_3_^–^) from the soil. Studies have shown that both the TF HY5 and its homolog HYH may directly induce the expression of the light-mediated high-affinity nitrate transporter gene *NRT2.1* and further activate nitrate transporters by promoting sucrose production and efflux (Chen et al., 2016). In view of this, we speculated whether *AtTPPI* could regulate primary root growth at high concentrations of nitrate.

**Figure 6:**
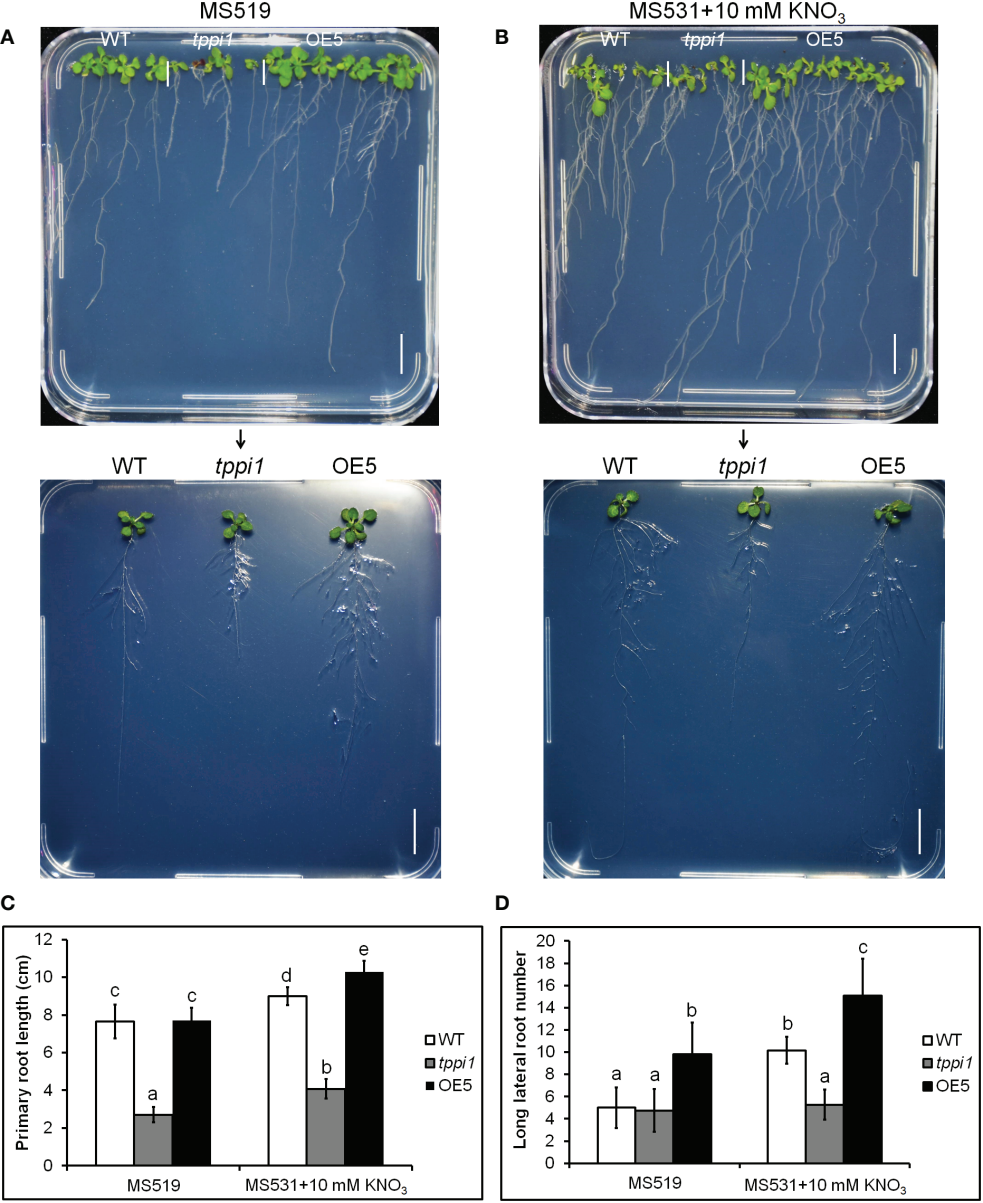
*AtTPPI* increase lateral root number under high nitrate condition. **(A, B)** The phenotype of WT, *tppi1* mutant, OE5 plant on MS vertical medium (MS519, **A**) and MS vertical nitrogen-deficiency medium (MS531+10 mM KNO_3_, **B**) for two-week days, Bar = 1 cm; **(C)** The primary root length statistical analysis of WT, *tppi1* mutant, OE5 plant on MS vertical total nitrogen medium (MS519) and MS vertical nitrogen free medium with 10 mM KNO_3_ (MS531+10 mM KNO_3_) for two-week days. Error bars indicate SEs (n = 3). Different lowercase letters **(a–e)** indicate significant differences among lines (Duncan’s test; p < 0.05); **(D)** The lateral root number statistical analysis of WT, *tppi1* mutant, OE5 plant on MS vertical total nitrogen medium (MS519) and MS vertical nitrogen free medium with 10 mM KNO_3_ (MS531+10 mM KNO_3_) for two-week days. Error bars indicate SEs (n = 3). Different lowercase letters **(a–e)** indicate significant differences among lines (Duncan’s test; p < 0.05).

Above transcriptome data also found that, there were two downregulated genes, *DEG18* and *AIR1* (**Figure 3B**). *DEG18* (which is also named *Araport11*) is an auxin-responsive family protein-encoding gene that may function in lateral root formation and be activated by *OXS2* under salt treatment (Neuteboom et al., 1999; Jing et al., 2019). *AIR1* is an auxin-induced gene and may also participate in lateral root formation (Neuteboom et al., 1999). Therefore, we further explore the lateral root phenotypes of the *tppi1* mutant and WT plants. The results showed that after 14 days of growth on nitrogen free medium with 10 mM KNO_3_ (MS531+10 mM KNO_3_), the OE5 plants still had significantly longer primary roots than the WT plants and *tppi1* mutants did (**Figure 6B**). On the control MS media, the primary root length of the OE plants was not different from that of the WT (**Figure 6A**). Further statistical analysis confirmed the above results (**Figure 6C**). In addition, we found that the number of lateral root number of OE5 was significantly more than the WT plant under high nitrate conditions (**Figure 6D**), and the *tppi1* mutant has less lateral root number than WT plant. The statistical analysis also proved that (**Figure 6D**). Different from the results of primary root growth, under the total nitrogen medium (MS519), the OE5 plants also has significantly more lateral root number (**Figure 6D**), while the *tppi1* mutant has same phenotype with the WT plants.

### 3.5 *AtTPPI* regulate lateral root number and elongation

**Figure 7:**
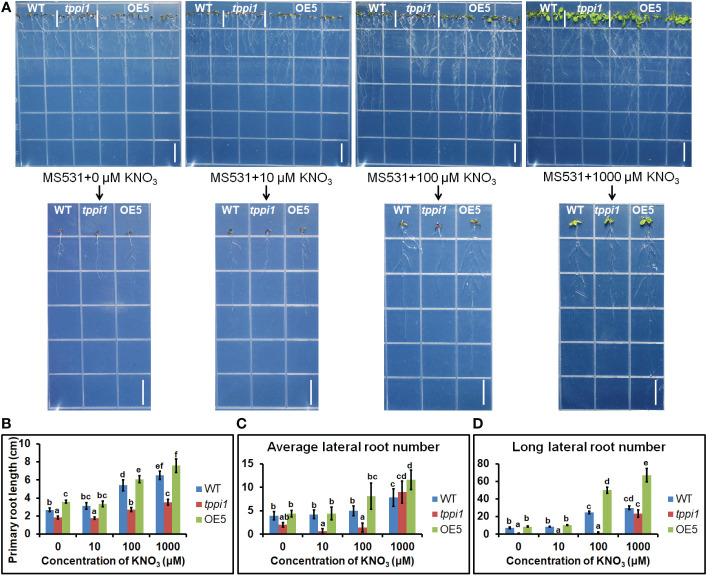
*AtTPPI* increase lateral root number and promote its elongation. **(A)** The phenotype of WT, *tppi1* mutant, OE5 plant on MS vertical nitrogen-deficiency medium with different concentration of KNO_3_ (0, 10 μM, 100 μM and 1000 μM) for 12 days, the graph above shows the overall results and the graph below shows the phenotypes of individual representative seedlings, Bar = 1cm; **(B)** The primary root length statistical analysis of WT, *tppi1* mutant, OE5 plant on MS vertical nitrogen-free medium with different concentration of KNO_3_ (0, 10 μM, 100 μM and 1000 μM) for 12 days. Error bars indicate SEs (n = 3). Different lowercase letters **(A–F)** indicate significant differences among lines (Duncan’s test; p < 0.05); **(C)** The average lateral root number of WT, *tppi1* mutant, OE5 plant on MS vertical nitrogen-free medium with different concentration of KNO_3_ (0, 10 μM, 100 μM and 1000 μM) for 12 days. **(D)** The long lateral root number of WT, *tppi1* mutant, OE5 plant on MS vertical nitrogen-deficiency with different concentration of KNO_3_ (0, 10 μM, 100 μM and 1000 μM) for 12 days. The total number of seedlings counted was 14.

To further evaluate the effluence of *AtTPPI* on lateral root in detail, we performed the root assay under nitrogen free medium with different concentration of nitrate including 0, 10 μM, 100 μM and 1000 μM KNO_3_. The results showed that the primary root length of the *tppi1* mutant was significantly inhibited at all concentrations (**Figures 7A, B**). OE5 overexpression plants significantly increased the primary root length at the other three concentrations except for the 10 μM KNO_3_ condition (**Figures 7A, B**). These results indicated that the application of KNO_3_ promoted the growth of primary roots. At the same time, the number and length of lateral roots were counted. The results showed that at 0, 10, 100 μM, the average lateral root number of the *tppi1* mutant was significantly lower than that of the wild-type plant, while at 1000 μM, the average lateral root number of the *tppi1* mutant and OE5 was higher than that of the wild-type plant (**Figure 7C**). In addition, at the concentration of 100 and 1000 μM KNO_3_, the long lateral root number of OE5 plants was significantly higher than the *tppi1* mutants and WT plants (**Figure 7D**). Combined the above results, it can be seen that exogenous potassium nitrate can promote not only the growth of primary root, but also the growth and elongation of lateral root.

## 4 Discussion

Our previous studies have demonstrated that Arabidopsis mutants with the mutated *AtTPPI* gene, *tppi1*, have impaired auxin transport, which in turn affects the cells of the elongation zone of the primary root (Lin et al., 2020). In addition, the PIN1 and PIN3 protein levels were also decreased in the *tppi1* mutant, which indicates that the *AtTPPI* gene may influence PIN1 and PIN3 through a certain method. PIN1 plays a direct role in polar auxin flow in the stele (Blilou et al., 2005; Feraru and Friml, 2008). PIN3 also localizes to the inner lateral side of root pericycle cells (Feraru and Friml, 2008). The decreased PIN1 and PIN3 protein levels might indicate that polar and acropetal auxin transport is impaired in the *tppi1* plants. Additionally, auxin-dependent phosphorylation of PIN3 polarization may be involved in maintaining high auxin levels in the vasculature near wound sites during root regeneration (Bustillo-Avendaño et al., 2018). What are the actual effect changes in the *AtTPPI* gene on the auxin content of plants? In this study, we found that the *tppi1* mutants indeed have reduced auxin content, which is consistent with our previous findings. When the expression of *AtTPPI* was elevated, the content of auxin was significantly increased compared to that in the WT plants (**Figure 2**), indicating that the *AtTPPI* gene likely acts as a positive regulator to regulate the content of auxin.

In addition, the results of our transcriptomic data analysis also showed that some key genes were related to auxin signaling. Several genes belonging to the GH3 family, including *GH3.3*, *GH3.9* and *GH3.12*, were significantly upregulated in the *tppi1* mutants. GH3 genes were the major auxin-responsive genes, and they are kinds of early auxin-responsive genes (Hooykaas et al., 1999). GH3 proteins have auxin aminase activity, which can mediate the conjugation of IAA to amino acids and thus inactivate auxin to maintain the dynamic balance of auxin in plants [50]. Therefore, changes in the expression of *GH3* genes can modulate plant development and auxin homeostasis [50]. *GH3.3* and *GH3.9* belong to the group II GH3 family, and their products catalyze the adenylation of IAA to amino acids (Staswick, 2005; Rachel and Mary, 2011). Therefore, it is likely that the increased expression levels of *GH3.3* and *GH3.9* caused the decreased auxin level in the *tppi1* mutant. The presence of these genes indicated that the *AtTPPI* gene might modulate auxin levels by directly or indirectly influence the expression of these genes. We speculated that PIN3 phosphorylation and increased expression levels of *GH3.3* and *GH3.9* may jointly cause the low auxin level of the *tppi1* mutant.

Moreover, as a member of the group III GH3 family, *GH3.12* was discovered to participate in the conjugation of SA to amino acids and may play a role in the plant defense response. The expression of *GH3.12* is induced by SA, and GH3.12 can use benzoate, an SA analog, as a substrate (Wang et al., 2011). Recently, GH3.12 (PBS3) was discovered to conjugate isochorismatase with glutamate to produce isochorismatase-glutamate, which is nonenzymatically and spontaneously converted into SA (Wang et al., 2011). Among the 7 up-regulated genes, there was a cytochrome p450 enzyme family gene *CYP83A1* (cytochrome P450 83A1 monooxygenase). A previous study found that *cyp83a1* mutants were resistant to powdery mildew (Simu et al., 2016; Sherp et al., 2018), so the upregulated expression of *CYP83A1* in the *tppi1* mutant suggests that the *tppi1* mutant may be sensitive to powdery mildew and that the *AtTPPI* gene may participate in the defense response.

Our previous study also confirmed that auxin synthesis was not affect, and polar auxin transport was most likely affect. Fortunately, we found a gene involved in polar auxin transport, *5PTASE11*. *5PTASE11* is a member of the 5PTases (At5PTases) (Weis et al., 2014). At5PTases have been shown to be important components of the plant phosphatidylinositol phosphate (PtdInsP) signaling pathway by selectively utilizing inositol-containing secondary messengers (Berdy, 2001; Carland and Nelson, 2004). Wang et al. found that a deficiency in *5PTase13* resulted in increased PIN2 expression in the roots and altered vesicle transport (Zhong, 2004). The researchers also found that the *5PTase13* mutant showed reduced BFA (the vesicle trafficking inhibitor) inhibition of the cycling of PIN1 and PIN2 proteins, which was accompanied by the accelerated recycling of basal targeted PIN proteins to the plasma membrane (Lin et al., 2009). These findings suggest that *5PTase13* has a negative effect on the cycling of PIN proteins and a negative influence on auxin transport (Lin et al., 2009). The *At5PTase11* gene encodes a PtdInsP-specific 5PTase, and its hydrolysis substrates are phosphatidylinositol 4,5-phosphate (PtdIns(4,5)P2), PtdIns(3,5)P2, and PtdIns(3,4,5)P3 (Ercetin and Gillaspy, 2004). When plants respond to external stimuli, PtdIns(4,5)P2 can be hydrolyzed into soluble inositol 1,4,5-trisphosphate (Ins(1,4,5))P3 by phospholipase C (PLC). Ins(1,4,5)P3 can activate the release of Ca^2+^ from intracellular calcium pools, and as a secondary messenger, Ca^2+^ participates in the signal transduction pathway in plants (Ercetin and Gillaspy, 2004). In addition, Ins(1,4,5)P3 and Ca^2+^ have been reported to function as modifiers of cell polarity and polar auxin transport by modulating ectopic basal PIN1 polarity and further influencing the auxin level (Zhong, 2004). Regarding the decreased PIN1 protein level in the *tppi1* mutant, we hypothesize that this phenomenon may be due to the change in *5PTase11* that caused the hydrolyzation of PtdIns(4,5)P2 into Ins(1,4,5)P3 and further triggered the Ins(1,4,5)P3-mediated Ca^2+^ signal, influencing PIN1-dependent auxin transport. In the future, we will perform detailed research to confirm this hypothesis.

HY5, a well-known bZIP TF, was the first TF shown to be involved in promoting photomorphogenesis and has been extensively studied. HY5 has been implicated as a negative regulator of the auxin signaling pathway (Meijer and Munnik, 2003). PIN1 plays a role in direct polar auxin transport in the stele (Blilou et al., 2005; Feraru and Friml, 2008). Regarding the decreased PIN1 level in the *tppi1* mutant, we hypothesize that HY5 may negatively regulate PIN1 and further influence auxin polar transport. Apart from the modulation of auxin signaling, HY5 has been reported to induce the expression of the light-mediated high-affinity nitrate transporter gene *NRT2.1* and further activate nitrate transporters by promoting sucrose production and efflux (Chen et al., 2016). Our research results also showed that elevated *AtTPPI* led to enhanced primary root length under relative low nitrogen conditions. This probably occurred because HY5 tends to inhibit the expression of most nitrogen assimilation-related genes under low-nitrate conditions. Furthermore, we will research whether *AtTPPI*-mediated primary root growth under relative low nitrogen-deficient conditions is dependent on *NRT2.1*. For crop plants, the nitrogen metabolism pathway mediated by the HY5 light signal provides ideas for improving the nitrogen utilization efficiency of plants in agricultural production and promotes the second green revolution centered on the efficient utilization of nitrogen fertilizers.

Overall, based on the above findings, we developed a hypothetical response model for the *AtTPPI* gene promoting primary root growth. Once the HY5 TF is active, HY5 can directly modulate the expression of the *AtTPPI* gene and further trigger the expression of downstream genes regulated by *AtTPPI*. It is possible that these genes, including *GH3.3*, *GH3.9*, *GH3.12* and *5Ptase11*, may jointly maintain the dynamic balance of auxin in plants and that auxin transport acts synergistically to promote the smooth transport of auxin, which in turn promotes primary root elongation. Elevated *AtTPPI* promote primary root growth and lateral root growth and elongation. This indicated that the overexpression of *AtTPPI* indeed has the ability to improve plant primary root growth under relative low nitrate condition, which will provide new ideas for improving the nitrogen utilization efficiency of crop plants in agricultural production.

## Data availability statement

The original contributions presented in the study are included in the article/**Supplementary Material**. Further inquiries can be directed to the corresponding authors.

## Ethics statement

The wild-type (Col-0) used in this study was purchased from ABRC. The overexpression lines OE5 were constructed by our library using dip flower infection. We ensure that our experimental research on plants, including the collection of plant material, comply with relevant institutional, national and international guidelines and legislation.

## Author contributions

SQ and JJ jointly design the study and wrote this article, QL and JG did the most experimental part of the article, and ZZ did the RNA-seq data analysis. ZM, JW, SW, XG, JS did the data statically analysis, LK gave suggestions and some revisions to the article. YJ, TW, NY, YW performed the experiments on the effects of different concentrations of nitrate on the growth of primary and lateral roots of *Arabidopsis thaliana*. All authors contributed to the article and approved the submitted version.

## References

[B1] Adamowski M. Friml J. (2015). PIN-dependent auxin transport: Action, regulation, and evolution. Plant Cell 27 (1), 20. doi: 10.1105/tpc.114.134874 25604445 PMC4330589

[B2] Band L. R. Wells D. M. Fozard J. A. Ghetiu T. French A. P. Pound M. P.. (2014). Systems analysis of auxin transport in the arabidopsis root apex. Plant Cell 26 (3), 862–875. doi: 10.1105/tpc.113.119495 24632533 PMC4001398

[B3] Berdy S. E. (2001). Molecular characterization of At5PTase1, an inositol phosphatase capable of terminating inositol trisphosphate signaling. Plant Physiol. 126 (2), 801–810. doi: 10.1104/pp.126.2.801 11402208 PMC111170

[B4] Blilou I. Xu J. Wildwater M. Willemsen V. Paponov I. Friml J.. (2005). The PIN auxin efflux facilitator network controls growth and patterning in arabidopsis roots. Nature 433 (7021), 39–44. doi: 10.1038/nature03184 15635403

[B5] Busch A. Montgomery B. L. (2015). Interdependence of tetrapyrrole metabolism, the generation of oxidative stress and the mitigative oxidative stress response. Redox Biol. 4 (C), 260–271. doi: 10.1016/j.redox.2015.01.010 25618582 PMC4315935

[B6] Bustillo-Avendaño E. Ibáñez S. Sanz O. Barros J. S. Gude I. Perianez-Rodriguez J.. (2018). Regulation of hormonal control, cell reprogramming and patterning during *De novo* root organogenesis. Plant Physiol. 176 (2), 1709. doi: 10.1104/pp.17.00980 29233938 PMC5813533

[B7] Carland F. M. Nelson T. (2004). COTYLEDON VASCULAR PATTERN2–mediated inositol (1,4,5) triphosphate signal transduction is essential for closed venation patterns of arabidopsis foliar organs. Plant Cell Online 16 (5), 1263–1275. doi: 10.1105/tpc.021030 PMC423214 15100402

[B8] Casimiro I. Beeckman T. Graham N. Bhalerao R. Zhang H. Casero P.. (2003). Dissecting arabidopsis lateral root development. Trends Plant Sci. 8 (4), 165–171. doi: 10.1016/S1360-1385(03)00051-7 12711228

[B9] Chen X. Yao Q. Gao X. Jiang C. Fu X. (2016). Shoot-to-Root mobile transcription factor HY5 coordinates plant carbon and nitrogen acquisition. Curr. Biol. Cb 26 (5), 640–646. doi: 10.1016/j.cub.2015.12.066 26877080

[B10] Cluis C. P. Mouchel C. F. Hardtke C. S. (2004). The arabidopsis transcription factor HY5 integrates light and hormone signaling pathways. Plant J. 38 (2), 332–347. doi: 10.1111/j.1365-313x.2004.02052.x 15078335

[B11] Ding Z. De Smet I. (2013). Localised ABA signalling mediates root growth plasticity. Trends Plant Sci. 18 (10), 533–535. doi: 10.1016/j.tplants.2013.08.009 24035235 PMC3791404

[B12] Ercetin M. E. Gillaspy G. E. (2004). Molecular characterization of an arabidopsis gene encoding a phospholipid-specific inositol polyphosphate 5-phosphatase. Plant Physiol. 135 (2), 938–946. doi: 10.1104/pp.104.040253 15181205 PMC514128

[B13] Feraru E. Friml J. (2008). PIN polar targeting. Plant Physiol. 147 (4), 1553–1559. doi: 10.1104/pp.108.121756 18678746 PMC2492634

[B14] Garay-Arroyo A. De La Paz Sánchez M. García-Ponce B. Azpeitia E. (2012). Hormone symphony during root growth and development. Dev. Dynamics 241 (12), 1867–1885. doi: 10.1002/dvdy.23878 23027524

[B15] Gelderen K. V. Kang C. K. Li P. Pierik R. (2021). Regulation of lateral root development by shoot-sensed far-red light *via* HY5 is nitrate-dependent and involves the NRT2.1 nitrate transporter. Front. Plant Sci 12. doi: 10.3389/fpls.2021.660870 PMC8045763 33868355

[B16] Hooykaas P. Hall M. A. Libbenga K. R. (1999). Biochemistry and Molecular Biology of Plant Hormones. Elsevier Science; 1 edition.

[B17] Huang H. Liu B. Liu L. Song S. (2017). Jasmonate action in plant growth and development. J. Exp. Bot. 6), 1349–1359. doi: 10.1093/jxb/erw495 28158849

[B18] Jing Y. Shi L. Li X. Zheng H. Zhang W. (2019). OXS2 is required for salt tolerance mainly through associating with salt inducible genes, CA1 and Araport11, in arabidopsis. Sci. Rep. 9 (1), 20341. doi: 10.1038/s41598-019-56456-1 31889067 PMC6937310

[B19] Keek P. Skpa P. Libus J. Naramoto S. Tejos R. Friml J.. (2009). The PIN-FORMED (PIN) protein family of auxin transporters. Genome Biol. 10 (12), 1–11. doi: 10.1186/gb-2009-10-12-249 PMC2812941 20053306

[B20] Khan S. Stone J. M. (2007). Arabidopsis thaliana GH3.9 influences primary root growth. Planta 226 (1), 21–34. doi: 10.1007/s00425-006-0462-2 17216483

[B21] Kim D. Langmead B. Salzberg S. L. (2015). HISAT: A fast spliced aligner with low memory requirements. Nat. Methods 12 (4), 357–360. doi: 10.1038/nmeth.3317 25751142 PMC4655817

[B22] Lee J. He K. Stolc V. Lee H. Figueroa P. Gao Y.. (2007). Analysis of transcription factor HY5 genomic binding sites revealed its hierarchical role in light regulation of development. Plant Cell Online 19 (3), 731–749. doi: 10.1105/tpc.106.047688 PMC1867377 17337630

[B23] Lejay L. Tillard P. Lepetit M. Olive F. D. Filleur S. Daniel-Vedele F.. (1999). Molecular and functional regulation of two NO3- uptake systems by n- and c-status of arabidopsis plants. Plant J. 18 (5), 509–519. doi: 10.1046/j.1365-313X.1999.00480.x 10417701

[B24] Lin W. H. Chen X. Xue H. W. (2009). The role of arabidopsis 5PTase13 in root gravitropism through modulation of vesicle trafficking. Cell Research: English Version 10), 1191–1204. doi: 10.1038/CR.2009.105 19736566

[B25] Lin Q. Wang S. Yihang D. Wang J. Wang K. (2020). Arabidopsis thaliana trehalose-6-phosphate phosphatase gene TPPI enhances drought tolerance by regulating stomatal apertures. J. Exp. Bot 71, (4285–4297). doi: 10.1093/jxb/eraa173 32242234

[B26] Luo J. Havé M. Clement G. Tellier F. Masclaux-Daubresse C. (2020). Integrating multiple omics to identify common and specific molecular changes occurring in arabidopsis under chronic nitrate and sulfate limitations. J Exp Bot, 71 6471-6490. doi: 10.1093/jxb/eraa337 32687580

[B27] Malamy J. E. (2010). Intrinsic and environmental response pathways that regulate root system architecture. Plant Cell Environ. 28 (1), 67–77. doi: 10.1111/j.1365-3040.2005.01306.x 16021787

[B28] Mariana R. Javier P. (2011). Salicylic acid beyond defence: its role in plant growth and development. J. Exp. Bot. 10), 3321. doi: 10.1093/jxb/err031 21357767

[B29] Meijer H. Munnik T. (2003). Phospholipid-based signaling in plants. Annu. Rev. Plant Biol. 54 (1), 265–306. doi: 10.1146/annurev.arplant.54.031902.134748 14502992

[B30] Murphy A. S. (2011). Seven things we think we know about auxin transport. Mol. Plant 4 (3), 487–504. doi: 10.1093/mp/ssr034 21505044

[B31] Nawkar G. M. Kang C. H. Maibam P. Park J. H. Jung Y. J. Chae H. B.. (2017). HY5, a positive regulator of light signaling, negatively controls the unfolded protein response in arabidopsis. Proc. Natl. Acad. Sci. United States America 114 (8), 2084. doi: 10.1073/pnas.1609844114 PMC5338426 28167764

[B32] Neuteboom L. W. Ng J. M. Y. Kuyper M. Clijdesdale O. R. Hooykaas P. J. J. Zaal B. J. V. D. (1999). Isolation and characterization of cDNA clones corresponding with mRNAs that accumulate during auxin-induced lateral root formation. Plant Mol. Biol. 39 (2), 273–287. doi: 10.1023/A:1006104205959 10080694

[B33] Nibau C. Gibbs D. J. Coates J. C. (2008). Branching out in new directions: the control of root architecture by lateral root formation. New Phytol. 179 (3), 595–614. doi: 10.1111/j.1469-8137.2008.02472.x 18452506

[B34] Olatunji D. Geelen D. Verstraeten I. (2017). Control of endogenous auxin levels in plant root development. Int. J. Mol. Sci. 18 (12), 2587–2615. doi: 10.3390/ijms18122587 29194427 PMC5751190

[B35] Oyama T. Shimura Y. Okada K. (1997). The arabidopsis HY5 gene encodes a bZIP protein that regulates stimulus-induced development of root and hypocotyl. Genes Dev 11, (2983–2995). doi: 10.1101/gad.11.22.2983 9367981 PMC316701

[B36] Paponov I. A. Palme K. Teale W. D. (2013). Auxin in action: signalling, transport and the control of plant growth and development. Nat Rev Mol Biol 7(847-859). doi: 10.1038/nrm2020 16990790

[B37] Park J. E. Park J. Y. Kim Y. S. Staswick P. E. Jeon J. Yun J.. (2007). GH3-mediated auxin homeostasis links growth regulation with stress adaptation response in arabidopsis. J. Biol. Chem. 282 (13), 10036–10046. doi: 10.1074/jbc.M610524200 17276977

[B38] Petersson S. V. Johansson A. I. Kowalczyk M. Wang J. Y. Moritz T. Grebe M. (2009). An auxin gradient and maximum in the arabidopsis root apex shown by high-resolution cell-specific analysis of IAA distribution and synthesis. Plant Cell 21, (1659–1668). doi: 10.1105/tpc.109.066480 19491238 PMC2714926

[B39] Petricka J. J. Winter C. M. Benfey P. N. (2012). Control of arabidopsis root development. Annu. Rev. Plant Biol. 63 (1), 563–590. doi: 10.1146/annurev-arplant-042811-105501 22404466 PMC3646660

[B40] Rachel A.O. Mary C.W. (2011). Evolutionary history of the GH3 family of acyl adenylases in rosids. Plant Mol. Biol. 76 (6), 489–505. doi: 10.1007/s11103-011-9776-y 21594748

[B41] Sabatini S. Beis D. Wolkenfelt H. Murfett J. Guilfoyle T. Malamy J.. (2006). An auxin-dependent distal organizer of pattern and polarity in the root. Cardiovasc. Res 5, (463–472). doi: 10.1016/s0092-8674(00)81535-4 10589675

[B42] Sascha W. Elizabeth S. Jürgen K. (2020). Same same, but different: growth responses of primary and lateral roots. J. Exp. Bot. 8), 8. doi: 10.1093/jxb/eraa027 PMC7178446 31956903

[B43] Shani E. Weinstain R. Zhang Y. Castillejo C. Kaiserli E. Chory J.. (2013). “Gibberellins accumulate in the elongating endodermal cells of arabidopsis root,” in Proceedings of the National Academy of Sciences of the United States of America, Vol. 110. 4834–4839. 10.1073/pnas.1300436110 PMC3606980 23382232

[B44] Sherp A. M. Westfall C. S. Alvarez S. Jez J. M. (2018). Arabidopsis thaliana GH3.15 acyl acid amido synthetase has a highly specific substrate preference for the auxin precursor indole-3-butyric acid. J. Biol. Chem. 293 (12), jbc.RA118.002006. doi: 10.1074/jbc.RA118.002006 PMC5868247 29462792

[B45] Sibout R. Sukumar P. Hettiarachchi C. Holm M. Muday G. K. Hardtke C. S. (2006). Opposite root growth phenotypes of hy5 versus hy5 hyh mutants correlate with increased constitutive auxin signaling. PloS Genet. 2 (11), e202. doi: 10.1371/journal.pgen.0020202 17121469 PMC1657057

[B46] Simu L. Bartnikas L. M. Volko S. M. Ausubel F. M. Dingzhong T. (2016). Mutation of the glucosinolate biosynthesis enzyme cytochrome P450 83A1 monooxygenase increases camalexin accumulation and powdery mildew resistance. Front. Plant Sci. 7, 227. doi: 10.3389/fpls.2016.00227 26973671 PMC4774424

[B47] Smyth G. K. (2010). edgeR: a bioconductor package for differential expression analysis of digital gene expression data. Bioinformatics 26 (1), 139. doi: 10.1093/bioinformatics/btp616 19910308 PMC2796818

[B48] Staswick P. E. (2005). Characterization of an arabidopsis enzyme family that conjugates amino acids to indole-3-acetic acid. Plant Cell 17 (2), 616–627. doi: 10.1105/tpc.104.026690 15659623 PMC548830

[B49] Toledo-Ortiz G. Johansson H. Lee K. P. Bou-Torrent J. Stewart K. Steel G.. (2014). The HY5-PIF regulatory module coordinates light and temperature control of photosynthetic gene transcription. PloS Genet. 10 (6), e1004416. doi: 10.1371/journal.pgen.1004416 24922306 PMC4055456

[B50] Wang Y. Jiao Y. L. (2018). Auxin and above-ground meristems. J. Exp. Bot. 69 (2), 147–154. doi: 10.1093/jxb/erx299 28992121

[B51] Wang G.-F. Seabolt S. Hamdoun S. Ng G. Park J. (2011). Multiple roles of WIN3 in regulating disease resistance, cell death, and flowering time in arabidopsis. Plant Physiol 156, (1508–1519). doi: 10.1104/pp.111.176776 21543726 PMC3135961

[B52] Warren M. J. Smith A. G. (2009). Tetrapyrroles: birth, life, and death tetrapyrroles: birth, life, and death. Landes Bioscience, Springer Science and Business Media

[B53] Wei Z. Jia L. (2016). Brassinosteroids regulate root Growth,Development, and symbiosis. Mol. Plant (English Version) 9 (1), 86–100. doi: 10.1016/j.molp.2015.12.003 26700030

[B54] Weis C. Hildebrandt U. Hoffmann T. Hemetsberger C. Pfeilmeier S. König C.. (2014). CYP83A1 is required for metabolic compatibility of arabidopsis with the adapted powdery mildew fungus erysiphe cruciferarum. New Phytol. 202 (4), 1310–1319. doi: 10.1111/nph.12759 24602105

[B55] Weiste C. Dröge-Laser W. (2014). The arabidopsis transcription factor bZIP11 activates auxin-mediated transcription by recruiting the histone acetylation machinery. Nat. Commun. 5, 1–12. doi: 10.1038/ncomms4883 24861440

[B56] Xu P. Zhao P. X. Cai X. T. Mao J. L. Xiang C. B. (2020). Integration of jasmonic acid and ethylene into auxin signaling in root development. Front. Plant Sci. 11. doi: 10.3389/fpls.2020.00271 PMC7076161 32211015

[B57] Zazimalova E. Murphy A. S. Yang H. Hoyerova K. Hosek P. (2010). Auxin transporters–why so many? Cold Spring Harbor Perspect. Biol. 2 (3), a001552. doi: 10.1101/CSHPERSPECT.A001552 PMC2829953 20300209

[B58] Zhong R. (2004). FRAGILE FIBER3, an arabidopsis gene encoding a type II inositol polyphosphate 5-phosphatase, is required for secondary wall synthesis and actin organization in fiber cells. Plant Cell Online 16, (3242–3259). doi: 10.1105/tpc.104.027466 PMC535871 15539468

